# Correlation between the Density of *Acipenser sinensis* and Its Environmental DNA

**DOI:** 10.3390/biology13010019

**Published:** 2023-12-28

**Authors:** Xiaojing Wang, Guangpeng Feng, Jiazhi Zhu, Wei Jiang

**Affiliations:** 1 East China Sea Fisheries Research Institute, Chinese Academy of Fishery Sciences, Shanghai 200090, China; wxj1212youxiang@163.com; 2 College of Ecology and Environment, Anhui Normal University, Wuhu 241002, China; 3 Hubei Key Laboratory of Three Gorges Project for Conservation of Fishes, Chinese Sturgeon Research Institute, China Three Gorges Corporation, Yichang 443100, China; zhu_jiazhi@ctg.com.cn (J.Z.); jiang_wei6@ctg.com.cn (W.J.)

**Keywords:** eDNA, density, *Acipenser sinensis*, relevance, degradation, qPCR

## Abstract

**Simple Summary:**

The objective of this study was to investigate the correlation between juvenile *Acipenser sinensis* density and the eDNA content in a controlled laboratory aquarium environment. The results obtained from fluorescent quantitative PCR indicated an overall increasing trend in the eDNA content, which tended to stabilize after three days. Furthermore, a significant positive linear correlation was observed between the density of juvenile *A. sinensis* and eDNA content at different time points (*R*^2^ = 0.768~0.986). When the juvenile *A. sinensis* were removed at a density of 0.114 ind./L, there was a negative correlation between eDNA content and time. Based on *AIC* and *BIC* analyses, a power function model was employed to illustrate the degradation relationship between eDNA content and time.

**Abstract:**

Since the construction of the Gezhouba Dam, Chinese sturgeon (*Acipenser sinensis*) numbers have gradually declined, rendering this species critically endangered according to the International Union for the Conservation of Nature. Environmental DNA (eDNA) technology plays an important role in monitoring the abundance of aquatic organisms. Species density and biomass have been proven to be estimable by researchers, but the level of accuracy depends on the specific species and ecosystem. In this study, juvenile *A. sinensis*, an endangered fish, were selected as the research target. Under controlled laboratory conditions in an aquarium, one, two, four, six, and eight juvenile *A. sinensis* were cultured in five fish tanks, respectively. Water samples were filtered at eight different time points for eDNA content analysis. Additionally, eDNA yield was tested at six different time points after a 0.114 ind./L density of *A. sinensis* was removed, and the employed degradation model was screened using the Akaike information criterion (*AIC*) and the Bayesian information criterion (*BIC*). The results showed that eDNA content remained stable after 3 days and exhibited a significant positive linear correlation with the density of *A. sinensis* (*R*^2^ = 0.768~0.986). Furthermore, eDNA content was negatively correlated with the 3-day period after the removal of *A. sinensis*. The power function had the smallest *AIC* and *BIC* values, indicating better fitting performance. This study lays a momentous foundation for the application of eDNA for monitoring juvenile *A. sinensis* in the Yangtze Estuary and reveals the applicability of eDNA as a useful tool for assessing fish density/biomass in natural environments.

## 1. Introduction

The Chinese sturgeon (*Acipenser sinensis*), belonging to the order Acipenseriformes, the family Acipenseridae, and the genus *Acipenser*, is a unique, large river and sea migration fish in China. In the summer, breeding adult fishes migrate from the Yangtze Estuary to the upper reaches of the Yangtze River, stay for one year in the Yangtze River, and spawn during the next autumn [1]. Due to the interception of the Gezhouba Dam project and the impoundment of the Three Gorges Reservoir, the spawning area of *A. sinensis* has shifted, and its spawning habitat has been destroyed [2,3]. Wild *A. sinensis* numbers have decreased sharply; thus, *A. sinensis* has been listed as a critically endangered species, and its biomass has become even more difficult to assess. According to the bulletin of aquatic biological resources and habitat status in the Yangtze River basin in 2022, the natural spawning activities of *A. sinensis* were not detected in the Yichang River section of Hubei Province in the Yangtze River during the dissection of egg-predatory fishes, the observation of underwater videos, and the investigation of egg harvesting at the bottom of the river [4]. Even the natural reproduction of *A. sinensis* has been consecutively interrupted for 6 years since 2017 [4]. In addition, the breeding population of *A. sinensis* was estimated to be only 13 according to a hydroacoustic survey conducted in the Yichang River section below the Gezhouba Dam [4]. According to the 2022 bulletin, more than 310,000 *A. sinensis* individuals have been released in the Yangtze River basin. However, only 20 sub-adult *A. sinensis* individuals were discovered in the middle and lower reaches of the Yangtze River and the Yangtze River estuary, with no wild juvenile *A. sinensis* individuals observed [4]. No signs of spawning activity were detected in 2013 and 2014; however, Zhuang et al. reported four wild *A. sinensis* individuals in the Yangtze Estuary in 2015 [5], suggesting that certain individuals had resumed spawning at uncharted locations within the Yangtze River due to their remarkable environmental adaptability. Therefore, a comprehensive and persistent resource survey of *A. sinensis* is imperative.

It has recently become of paramount importance to investigate fishery resources, necessitating the adoption of less intrusive investigation methods. Traditional surveys such as bottom trawling and using fyke nets entail substantial human and material resource consumption [6,7] while also requiring taxonomic expertise, thereby posing a significant risk of ecosystem disturbance with respect to the riverbed. The publication of lists of banned fishing gear and the development of energy-efficient fishing gear have cut down on the widespread use of such survey methods and contributed to significant biodiversity improvements on the river bottom [8]. In addition, the tracking method of using a mark is complicated, costly, and damages fish; thus, the amount of tagging is limited [9]. Environmental DNA (eDNA) technology has supplied a new idea and method for the investigation of aquatic organisms. It makes up for the shortcomings of the above survey methods. The skin, mucus, tissue, feces, and urine shed and excreted by organisms are together referred to as environmental DNA (eDNA), which was first used to monitor traces of hydrobiont in 2008 [10]. eDNA technology has been extensively applied to monitor multiple species and estimate biodiversity in various aquatic ecosystems [11]. Compared to traditional investigation methods, eDNA technology offers faster, simpler, and more sensitive and efficient approaches. Additionally, it is less invasive, and it is particularly effective for monitoring low-natural-density, endangered, and rare species [12,13]. Yu et al. proved that environmental DNA was effective in monitoring the breeding populations of *A. sinensis* [14], and Xu et al. utilized eDNA to monitor the seasonal spawning pattern of *A. sinensis* [15], both of which highlighted the feasibility and significance of further studying eDNA technology for monitoring *A. sinensis*.

Recently, scholars have also adopted eDNA methods to estimate species biomass, density, and abundance [16]. Several studies have demonstrated positive correlations between species biomass and eDNA concentration [17,18,19]. Takahara et al. discovered a positive correlation between the biomass of carp and eDNA concentrations in both aquariums and ponds [20]. Itakura et al. estimated the abundance and biomass of eel through eDNA analysis in river basins [21]. An increasing number of studies have linked eDNA concentrations with species density/biomass [22,23]. The shedding and degradation rate of eDNA is affected by the differences in species, which also have an impact on the correlation between concentration and density. *A. sinensis* is an endangered migratory fish that inhabits complex river and sea environments. However, the research on the relationship between eDNA content and the density of *A. sinensis* is limited.

Thus, the aim of this study was to preliminarily investigate the relationship between eDNA content and density in a controlled environment using a fluorescence quantitative PCR technique. Moreover, we also conducted research on the degradation of eDNA content over time after removing eight juvenile *A. sinensis* to establish an appropriate model for the relationship between the eDNA content of *A. sinensis* and time. This was carried out by using the Akaike information criterion (*AIC*) and the Bayesian information criterion (*BIC*). This study analyzed the correlation between the density of *A. sinensis* (biomass) and the concentration of eDNA. These results will provide valuable insights into density assessment and resource distribution monitoring in natural habitats while offering reliable data for conservation efforts focused on aquatic biological resources.

## 2. Materials and Methods

### 2.1. The Specific Primers and Probes for A. sinensis

The primer sequence used in this study for amplifying the 132 bp target fragment of the *D-loop* gene (GenBank accession number: AY096295.1) was designed by Yu et al. [14]. The primer sequence is as follows:

*D-loop*F (DF): GGCAATTTTAATCTGGGTTTCCA;

*D-loop*R (DR): TGGATGTTAGATATATGTCCTTG.

According to the mitochondrial DNA (mtDNA) *D-loop* gene sequence of *A. sinensis* (GenBank accession number: AY096295.1), the probe was designed by Shanghai Personal Biotechnology (Shanghai, China) Co., Ltd., and the probe sequence is as follows:

Probe: 5′FAM-CAAGGTAGAACATTACACAACTGCTCG-3′BHQ1.

Moreover, the primers and probe were validated using Primer BLAST of NCBI and checked for potential cross-amplifications in syntopic taxa [14].

### 2.2. Experiment Design and Sample Collection

The 70 L aquarium, equipped with the same filtration and aeration device, was utilized. Following a 24-h disinfection period using potassium permanganate solution, the aquarium was filled with filtered tap water and aerated for over 3 days. The quality parameters of water were monitored to maintain a temperature of 20 ± 1 °C, a pH of 7.69–8.01, a dissolved oxygen concentration of >7.5 mg/L, and a concentration of ammonia nitrogen of <0.2 mg/L. The light cycle followed a pattern of 12 h of light followed by 12 h of darkness (12L:12D). The F2 *A. sinensis* (approximately 0.6 years old) were artificially bred in October 2022. Five juvenile *A. sinensis* density treatments (1, 2, 4, 6, and 8 individuals in each tank) were set up. A total of 63 juvenile *A. sinensis* were randomly divided over 15 aquaria, and each treatment consisted of three replicates. Healthy juvenile *A. sinensis* with similar specifications were selected. The average full length of *A. sinensis* was (25.26 ± 0.78) cm; the average body length was (23.81 ± 0.39) cm; and the average body weight was (48.20 ± 0.83) g. Moreover, the aquaculture water was constantly recycled and filtered to maintain freshness. At 8:00, 11:00, 14:00, and 17:00, the total feeding amount was 10% of the fish’s body weight.

Experiment 1: In each of the five densities (0.014, 0.029, 0.057, 0.086, and 0.114 ind./L) of juvenile *A. sinensis*, 250 mL samples of water were collected from each tank at a depth of 10 cm below the water surface at the following time points: 0 h, 12 h, 1 d, 2 d, 3 d, 4 d, 5 d, and 7 d. Additionally, a negative control sample was taken from the blank aquarium.

Experiment 2: After collecting the water samples in experiment 1, all juvenile *A. sinensis* were removed to ensure a specific eDNA content in the water, thus preventing further release of eDNA by *A. sinensis* and minimizing the impact on the experimental results. Afterward, 250 mL samples of water were gathered at 0 h, 6 h, 12 h, 1 d, 2 d, and 3 d intervals only from three tanks with eight juveniles (0.114 ind./L). The negative control followed the same protocol as in experiment 1.

The water was filtered using mixed cellulose membranes with a pore size of 0.45 μm through a glass sand core vacuum device. After filtration, each filter membrane was individually packed into a 2 mL centrifuge tube and stored in a refrigerator at −80 °C until eDNA extraction. A filter cup was rinsed with ultrapure water three times before filtration. Cross-contamination and external contamination were avoided. The entire experimental operation was carried out in a sterilization environment on an ultra-clean table, with the researchers wearing protective clothing and sterile gloves and masks.

### 2.3. eDNA Extraction and PCR Amplification

The membrane samples, as described in Section 2.2, were cut into pieces using sterile tweezers and placed in a 2 mL centrifuge tube. A DNeasy Blood & Tissue Kit from Tiangen was used for eDNA extraction, according to the manufacturer’s instructions. After the extraction was accomplished, the eDNA concentration and purity (OD) were immediately detected via an ultraviolet spectrophotometer and stored at −20 °C.

The 25 μL PCR amplification system comprised 19.5 μL of ddH_2_O, 0.5 μL of forward and reverse primers, 2.5 μL of 10× buffer, 1.0 μL of mixed-template eDNA, 0.5 μL of dNTPs, 0.5 μL of Taq DNA polymerase. The PCR amplification profile included pre-denaturation at 95 °C for 5 min; denaturation at 95 °C for 30 s; annealing at 55 °C for 30 s; extension at 72 °C for 30 s (for a total of 35 cycles); maintaining elongation at 72 °C for 7 min; and storage at 4 °C. The PCR products were purified through 1.5% agarose gel electrophoresis, and then, the single and bright products of the electrophoresis bands were recovered using an AxyPrep-DNA gel Recovery kit.

### 2.4. Preparation of the Recombinant Plasmid Standard

The purified PCR product was ligated with pMD18-T plasmid vector. A volume of 1 μL of pMD18-T vector (50 ng/μL), 1 μL of Insert DNA, 3 μL of ddH_2_O, and 5 μL of Solution I were added into a tube and reacted at 4 °C for 1 day. A volume of 50 μL of DH5α receptive cell solution was added to 10 μL of the ligation product, and the mixture was then placed on ice for 0.5 h. Afterward, the mixture was heated in a 42 °C water bath for 90 s, and subsequently cooled on ice for 180 s. A volume of 700 μL of sterile 2YT medium was added and incubated at 37 °C for 45 min at 150 r/min in a shaker. Then, 100 μL of the bacterial solution was applied to 2YT solid medium (containing Amp) and inverted for 12–16 h at 37 °C. The bacterial solution of ligated *Escherichia coli* colonies was successfully selected for plasmid extraction, according to the instructions of E.Z.N.A^®^ Plasmid DNA Mini Kit I, which was performed in an aseptic manipulation environment.

After extracting the plasmid DNA, we measured the concentration and purity using a NanoDrop 2000 spectrophotometer and calculated the number of plasmid copies. Then, 10^10^–10^3^ copies/μL of 8 gradient templates were successively diluted three times with ddH_2_0, and the standard curve was constructed.

### 2.5. Amplification of Fluorescent Quantitative PCR

All eDNA solution samples, as described in Section 2.3, were tested by Shanghai Personal Biotechnology (Shanghai, China) Co., Ltd. The quantitative PCR (qPCR) reaction system consisted of 20 μL and was prepared as follows: 10 μL of 2× Probe real-time PCR premixture, 1.0 μL of DNA template, 0.2 μL of probe, 0.4 μL of forward primer, 0.4 μL of reverse primer, 8 μL of ddH_2_O. Quantstudio^TM^6 Flex PCR instrument was applied for qPCR amplification, the amplification profile includes pre-denaturation at 95 °C for 30 min, followed by 50 cycles of denaturation at 95 °C for 15 s and annealing at 60 °C for 30 s. Each eDNA sample, as described in Section 2.3, was repeated three times, with three positive and negative controls performed in each 96-well plate. The plasmid standard DNA described in Section 2.4 was diluted from 10^10^ copies/μL to 10^3^ copies/μL in a ten-fold concentration gradient for field use, which generated the standard curve. The experimental eDNA copies were analyzed using an absolute quantitative method and expressed as the average value and standard deviation.

### 2.6. Data Analysis

The mean and standard deviation of eDNA copies for each set of three samples were calculated using Excel 2023 software. Linear regression was used to analyze the relationship between eDNA contents and juvenile *A. sinensis* density using data from samples taken in experiment 1. Appropriate degradation models were screened using the Akaike information criterion (*AIC*) and the Bayesian information criterion (*BIC*), using data from samples taken in experiment 2. The regression equation and the coefficient of determination (*R*^2^) were obtained by selecting the 95% confidence interval.

## 3. Results

### 3.1. Specificity Verification of Primers and Probe

The amplification curve described the dynamic progression of PCR, with the number of cycles plotted on the abscissa and the signal intensity of real-time fluorescence during the reaction plotted on the ordinate. The amplification curve can be divided into four periods: baseline period, exponential period, linear period, and plateau period.

**Figure 1:**
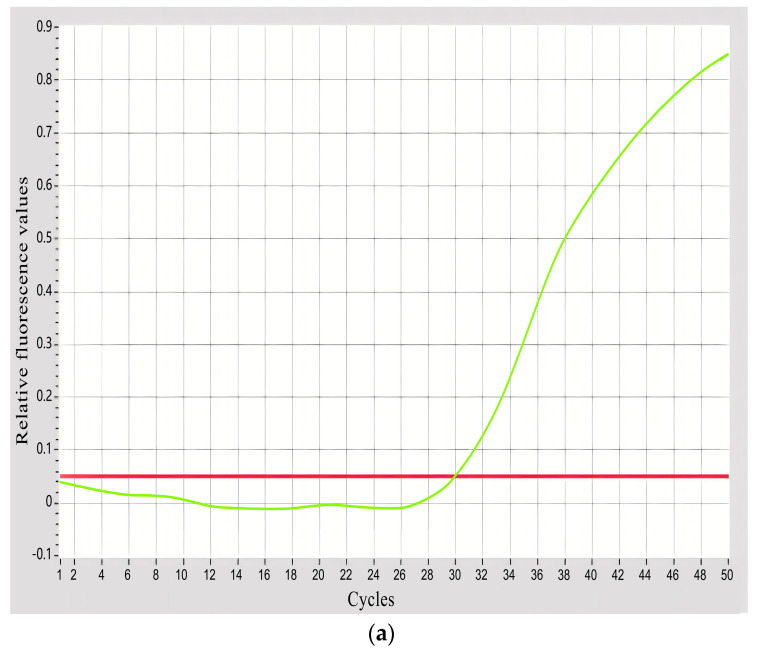

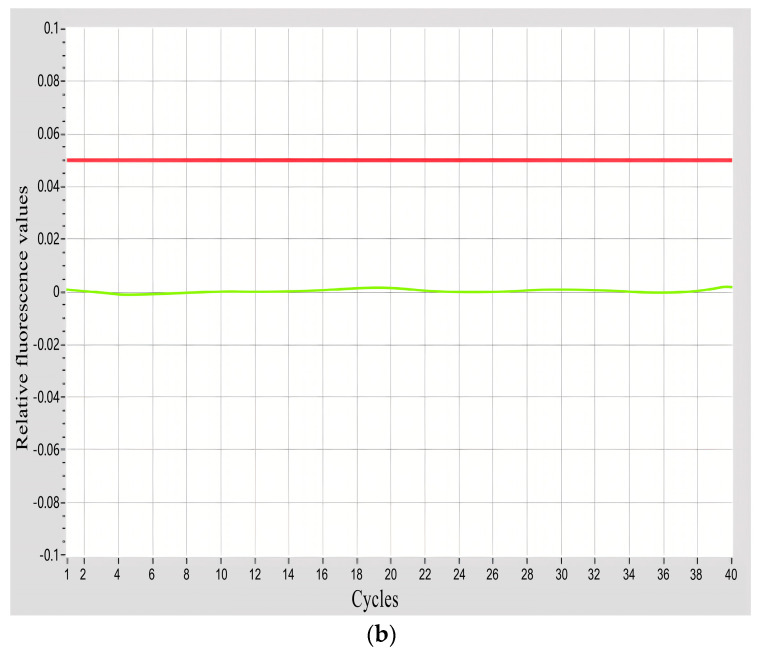
The amplification curve of *A. sinensis* specific primers. (**a**) Positive amplification. (**b**) Negative amplification. The red line represents the threshold line, while the green line depicts the amplification curve of the mixed sample. The CT value is determined via the intersection of these two lines.

After the amplification of fluorescence quantitative PCR utilizing the specific primers DF/DR and the probe of *A. sinensis* on all mixed experimental samples, four periods on the amplification curve were observed in the DNA extracted from all mixed experimental samples, indicating positive amplification. The fluorescence amplification curve only showed a baseline period in the blank control sample, indicating negative amplification (Figure 1). The results demonstrated the specificity of the primers and probe.

### 3.2. Establishment of Standard Curve and Regression Equation

**Figure 2:**
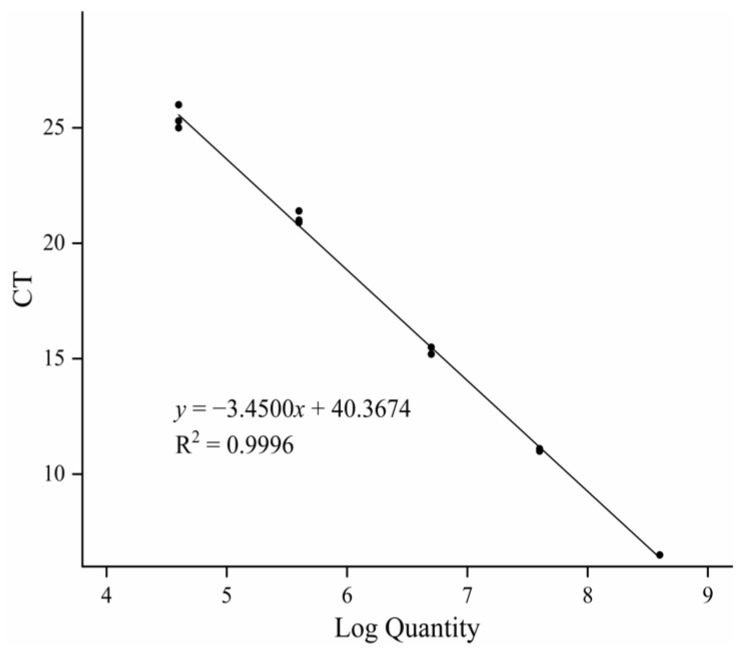
The standard qPCR curve of the mtDNA *D-loop* gene of *A. sinensis*.

The qPCR standard curve of the mtDNA *D-loop* gene of *A. sinensis* was generated by performing fluorescence quantitative PCR amplification, which involved systematic changes in CT values (Figure 2). The resulting correlation coefficient (*R*^2^) of the curve was 0.9996, and the slope (*K*) of the curve was −3.4500, yielding the regression equation of *y* = −3.4500*x* + 40.3674. The results showed a strong linear relationship within the range of diluted standard DNA concentrations, indicating that this standard curve could reliably reflect the amplification effect of the mtDNA *D-loop* gene of *A. sinensis*.

### 3.3. Changing Trend of eDNA Content of A. sinensis

**Figure 3:**
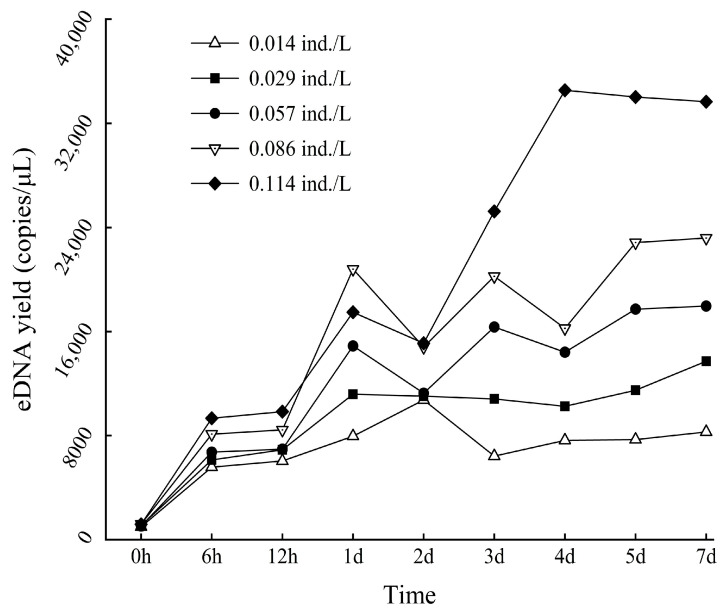
Variation trend of eDNA yield of *A. sinensis* cultured in lab tanks.

The quantitative detection was performed using specific primers for *A. sinensis*, and the eDNA content trends at different time points and densities were compared based on the mean number of copies. The eDNA content of *A. sinensis* changed significantly under laboratory conditions. The overall trend in the eDNA content of *A. sinensis* showed an initial increase over time, reaching a peak after 2 days and stabilizing after 3 days. Additionally, the overall trend in the eDNA content of *A. sinensis* increased with increasing density (Figure 3).

### 3.4. Correlation between eDNA Content and Density of A. sinensis

**Figure 4:**
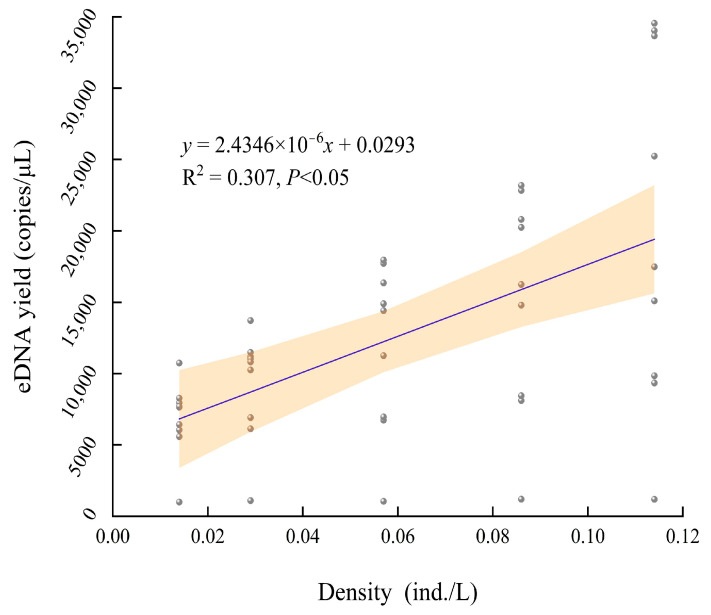
Correlation between the density of *A. sinensis* and the eDNA content from 0 h to 7 days.

**Figure 5:**
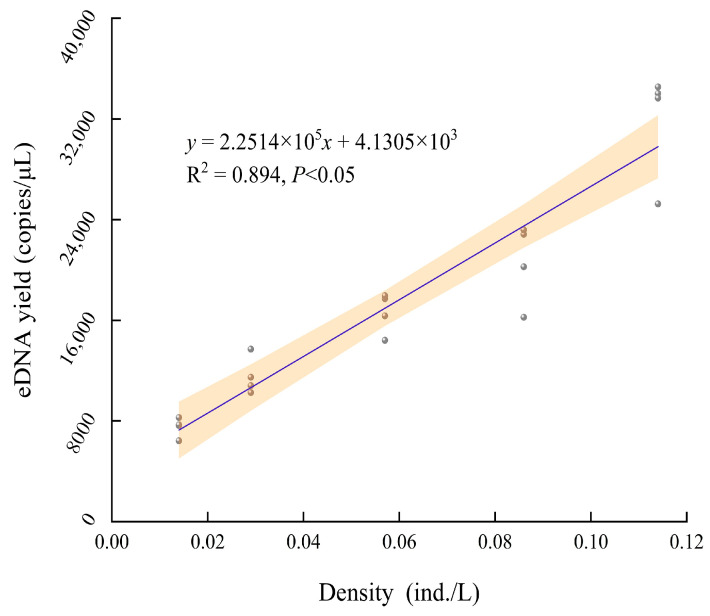
Correlation between the density of *A. sinensis* and the eDNA content from 3 days to 7 days.

By fitting the linear correlation between the eDNA content at different time points (0 h~7 d) and the five densities as a whole, a poor fitting effect and low correlation (*R*^2^ = 0.307) were discovered (Figure 4). A better fit (*R*^2^ = 0.894) was observed when integrally establishing the linear correlation between the stable eDNA content of 3 days to 7 days and the five densities (Figure 5). By separately fitting linear correlations between the eDNA content at the same time points (6 h~7 d) and different densities, the discovery of all better fitting effects was performed with the coefficient of determination approaching 1 (*R*^2^ = 0.768~0.986) (Table 1). These findings suggest a significant linear relationship between the density and the eDNA content of *A. sinensis*. As the density of *A. sinensis* increased, a higher number of eDNA copies were detected.

### 3.5. Degradation Results of eDNA of A. sinensis

**Figure 6:**
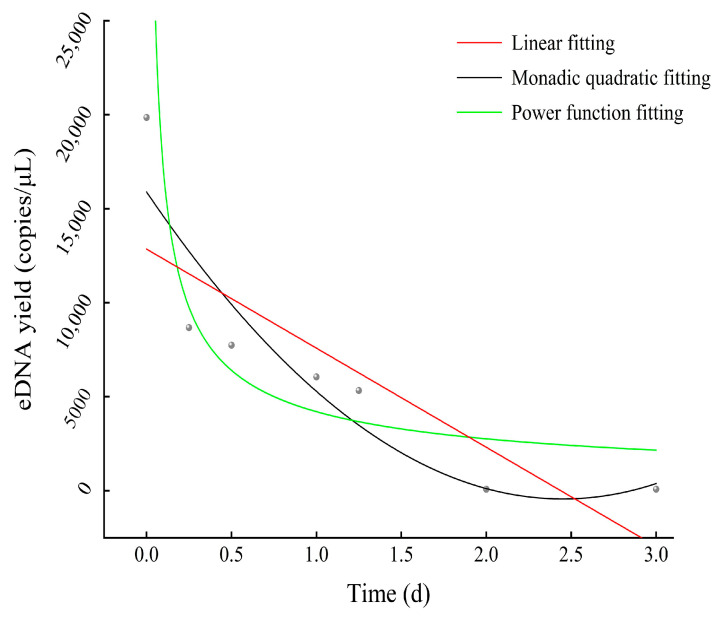
The degradation relationship between eDNA content and time based on three models.

After removing *A. sinensis* from the fish tank, this study investigated the degradation process of eDNA at a density of 0.114 ind./L. The findings revealed a negative correlation between the degradation of eDNA and time, with the most rapid degradation occurring within 0.25 days (*K* = −1862.31). The eDNA content measured on day zero was 19,851.67 copies/μL. Then, the eDNA content measured on the third day degraded to 82.81 copies/μL. The applicability of fitting the relationship between eDNA content and time was compared among linear function, monadic quadratic function, and power function based on *AIC* and *BIC* values. The results showed that the power function had the smallest *AIC* and *BIC* values (Table 2 and Figure 6). Therefore, the power function was selected to explain the degradation relationship between eDNA content and time. The formula for this is *y* = 4206.5738*x*^−0.6074^ (*R*^2^ = 0.706).

## 4. Discussion

### 4.1. Changes in eDNA Content

The eDNA content of *A. sinensis* with similar specifications exhibited an overall increasing trend under laboratory breeding conditions. This finding is consistent with the results reported by Karlsson et al. [24] and Sint et al. [25]. The eDNA content of *A. sinensis* detected at different densities increased within 0 h to 1 day, fluctuated between 1 and 3 days, and gradually stabilized after 3 days. Minamoto et al. observed a similar trend in the eDNA concentration of jellyfish (*Chrysaora pacifica*) during tank experiments, which tended to stabilize within 48 h [26]. However, Takahara et al. adopted environmental DNA to detect the eDNA concentration of *Cyprinus carpio* L. in an aquarium and discovered that the change in eDNA concentration was slow after 3 days and reached equilibrium after 6 days [20]. Thus, eDNA may be continuously released and degraded in water until the content or concentration reaches a plateau within three days, indicating an eventual balance between shedding and degradation processes. Although the initial content of eDNA at 0 h was very low across all five densities, it was not zero, which was due to the potential shedding from juvenile *A. sinensis* introduced into the tank environment. The fluorescent quantitative PCR method is appropriate for the detection of copy values at low concentrations, with a higher sensitivity in quantifying eDNA levels [26,27].

### 4.2. Correlation between eDNA Content and Density

The experiment explored the correlation between eDNA content and density using the qPCR method. A strong positive linear relationship was found between eDNA content and the density of juvenile *A. sinensis*, confirming that eDNA can be used as a reliable indicator for assessing species density or biomass. Recently, the use of eDNA technology to monitor rare and endangered species, economic species, and invasive species has become increasingly popular among scholars. Xin et al. discovered a significant positive correlation (*R*^2^ = 0.72~0.93) between the biomass of *Acanthopagrus latus* and eDNA concentrations at different time points [17]. Benoit et al. reported a positive linear correlation between the biomass of juvenile *Pacific salmon* and the eDNA concentrations detected at each sampling time point, except for 48 h [28]. The eDNA content of crayfish increased continually with an increase in density and time in aquaculture water, showing a positive linear correlation [25]. Takahara et al. investigated the variations in the eDNA concentration of carp in both aquarium and pond settings, revealing a significant positive correlation between the eDNA concentration and the biomass of carp [20]. A strong positive association was observed between the total biomass of *Octopus vulgaris* and the detected eDNA content in an aquarium [29]. The findings of this study are consistent with these conclusions. Generally, a linear correlation exists between eDNA content and fish density or biomass in still-water systems, such as fish tanks, pools, and ponds [30,31]. However, environmental factors, such as temperature, pH, and impurities, will lead to an uncertain and complicated relationship when dealing with large flowing bodies of water [32,33,34,35]. Pilliod et al. compared field investigations with environmental DNA analysis for populations of Rocky Mountain tailed frogs and Idaho giant salamanders, and they found that river density and biomass were positively correlated with the average eDNA concentration at low densities but not significantly related at high densities [36]. Pont et al. compared the results of a marine trawl survey with eDNA sampling data from New Jersey waters (USA), and discovered that the eDNA results highly coincided with the estimation of species richness and the relative abundance of marine fish species obtained through trawling [18]. Additionally, they observed a strong correlation between the number of eDNA species reads and the allometric growth index of species biomass [18]. By quantifying the population of *Salvelinus fontinalis* using eDNA from water samples collected from 40 streams, Baldigo et al. accurately predicted the existence and abundance of brook trout populations [37]. Environmental DNA and echo sounder techniques were adopted to survey the biomass of *Trachurus japonicus* in Maizuru Bay within the Sea of Japan. Yamamoto observed a significant positive linear correlation between the estimated eDNA concentration levels and echo intensity readings, which reflected fish biomass up to a range of 150 m [38]. These studies demonstrated that eDNA techniques can assess biomass in field conditions. Thus, juvenile *A. sinensis* with similar specifications and age were selected to conduct a preliminary exploration of the correlation between eDNA content and density. It is worth noting that the results obtained in controlled environments may not necessarily apply to natural ecosystems. The influence of biological and abiotic factors on eDNA content remains poorly understood in *A. sinensis*, which inhabits the complex bottom of rivers and seas, necessitating further verification.

### 4.3. Degradation of eDNA in Water

The shedding rates and degradation rates of organisms are related to the content of eDNA, which is affected by various biological and abiotic factors [34,39]. Once released into the water, large fragments of eDNA gradually decompose into small fragments over time until complete degradation occurs [40,41,42]. The degradation of eDNA is negatively correlated with time. Generally, when the release source is removed, shed eDNA can remain in the water body for 7~30 days. However, the size, amount, and persistence of shed eDNA fragments in the water varied among species [43,44]. The eDNA content of *Cyprinus carpio* was less than 5% after 96 days in a laboratory freshwater aquarium [45]. Goldberg et al. observed detectable levels of New Zealand *Potamopyrgus antipodarum* eDNA in aquariums, even after 21 days [46]. Due to the uncertainties and complexities in eDNA studies, the quantitative assessments of biomass are different among scholars. For instance, Barnes et al. and Goldberg et al. built an eDNA exponential decay model [45,46], while Cerco et al. employed a regression model to quantify the change in eDNA concentration [47]. This experiment only studied the degradation of eDNA in juvenile *A. sinensis* within 3 days, and found that the power function model based on *AIC* and *BIC* clarifies the relationship between degradation and time. Environmental factors such as temperature and pH had more influence on both the shedding and degradation processes related to eDNA [17,31,48]. In this study, stable water temperature, pH, and light conditions were maintained to minimize the potential impact of drastic changes on the degradation of eDNA content. *Procypris rabaudi*, an endangered fish species, exhibited a degradation time as long as 17 days [49]. Consequently, a detection method was established for assessing the biomass of *Procypris rabaudi* based on environmental DNA [49]. The persistence of eDNA fragments in water can provide valuable information regarding the presence or absence of species. Therefore, the degradation of eDNA is vital for studying rare and endangered species [50]. Environmental DNA technology represents an effective new approach for monitoring species, and plays a crucial part in the conservation of rare species when combined with traditional survey methods.

### 4.4. Biomass Assessment of eDNA

In recent years, more and more biomass assessments based on eDNA have been conducted. However, variations in species types, individual differences, and life history changes result in the differential release of eDNA, making accurate quantitative analysis challenging. Therefore, biomass assessment can be categorized into two types: laboratory-based and field-based assessments. Due to the extensive field water environment, laboratory simulations of ecological experiments are necessary to elucidate the correlation between the density of aquatic organisms and their environmental DNA [48], which will provide references for biomass assessment and establish a foundation for field biomass evaluation. This study preliminarily explicated the positive linear correlation between the eDNA content of juvenile *A. sinensis* and density under laboratory conditions. Additionally, this study observed a power function degradation process, indicating that eDNA technology is feasible for evaluating species biomass in the laboratory. Previous studies have also proven the feasibility of biomass assessment in wild environments [36,37,38]. However, due to water flow activities and environmental factors, these assessments may be affected [51]. As a result, it is necessary to combine environmental DNA technology with traditional investigation methods to provide a scientific basis and technical support for comprehensive biomass assessment.

## 5. Conclusions

Under controlled environmental conditions, this study preliminarily demonstrated a strong linear positive correlation between eDNA content and the density of juvenile *A. sinensis*. Furthermore, the study revealed that the removal time of *A. sinensis* was negatively correlated with its corresponding eDNA content over a period of 3 days following a power function model, suggesting gradual degradation. The findings highlighted the correlation between density and eDNA content, which can provide a reference for assessing the density of juvenile *A. sinensis* and demonstrate the applicability of environmental DNA in species monitoring of *A. sinensis*. This study established a theoretical basis for evaluating population resources of juvenile *A. sinensis*, and enhanced the understanding of population dynamics in migratory fish in rivers and seas.

## Figures and Tables

**Table 1:** Linear fitting of the density of *A. sinensis* and the eDNA content from 6 h to 7 days.

Time	Linear Fitting	*R*^2^ (*p* < 0.05) ^1^
6 h	*y* = 4955.51*x* + 529.96	0.983
12 h	*y* = 5511.27*x* + 508.16	0.936
1 d	*y* = 7953.28*x* + 1550.44	0.768
2 d	*y* = 9626.96*x* + 704.68	0.867
3 d	*y* = 4966.78*x* + 2583.90	0.986
4 d	*y* = 2455.37*x* + 3372.55	0.834
5 d	*y* = 3774.58*x* + 3566.41	0.979
7 d	*y* = 5354.20*x* + 3335.19	0.971

^1^ There were significant differences.

**Table 2:** The choice of model based on *AIC* and *BIC* values.

Items	Linear Function	Monadic Quadratic Function	Power Function
*AIC*	127.8203	137.0612	108.2885
*BIC*	119.6581	116.8449	95.6638

## Data Availability

The data presented in this study are available in the article. Further information is available upon request from the corresponding author.

## References

[1] Zhou C.S., Xu Y.G., Deng Z.L., Yu Z.T. (1985). Observation on the reproductive glands of adult *Acipenser sinensis* gray in Changjiang River below Gezhouba Dam. Acta Hydrobiol. Sin..

[2] Gao X., Brosse S., Chen Y.B., Lek S., Chang J.B. (2009). Effects of damming on population sustainability of Chinese sturgeon, *Acipenser sinensis*: Evaluation of optimal conservation measures. Environ. Biol. Fishes.

[3] Gao X., Lin P.C., Li M.Z., Duan Z.H., Liu H.Z. (2016). Impact of the Three Gorges Dam on the spawning stock and natural reproduction of Chinese sturgeon in Changjiang River, China. Chin. J. Oceanol. Limnol..

[4] Zhuang P., Zhao F., Zhang T., Chen Y., Liu J.Y., Zhang L.Z., Kynard B. (2016). New evidence may support the persistence and adaptability of the near-extinct Chinese sturgeon. Biol. Conserv..

[5] Yangtze River Basin Fishery Administration Office of the Ministry of Agriculture and Rural Affairs, the Changjiang Water Resources Commission of the Ministry of Water Resources, the Yangtze River Basin ecological Environment Supervision Administration of the Ministry of Ecology and Environment, The Changjiang River Adminstration of Navigational Affairs, Mot (2022). The Bulletin of Aquatic Biological Resources and Habitat Status in the Yangtze River Basin.

[6] Bohmann K., Evans A., Gilbert M.T.P., Carvalho G.R., Creer S., Knapp M., Yu D.W., Bruyn M.D. (2014). Environmental DNA for wildlife biology and biodiversity monitoring. Trends Ecol. Evol..

[7] Anish K., Daniel W., Thomas N., Liza B., Claire O., Riley P., Ashley C., Katie L., Lauren S. (2021). Quantification of Environmental DNA (eDNA) shedding and decay rates for three commercially harvested fish species and comparison between eDNA detection and trawl catches. Environ. DNA.

[8] Mytilineou C., Herrmann B., Smith C.J., Mantopoulou P.D., Anastasopoulou A., Siapatis A., Sala A., Megalofonou P., Papadopoulou N., Vassilopoulou V. (2022). Impacts on biodiversity from codend and fisher selection in bottom trawl fishing. Front. Mar. Sci..

[9] Zhou H.X., Gan W.X. (2017). Advances in fish labeling technology and its application in artificial proliferation and release. Hubei Agric. Sci..

[10] Ficetola G.F., Miaud C., Pompanon F., Taberlet P. (2008). Species detection using environmental DNA from water samples. Biol. Lett..

[11] Rourke M.L., Fowler A.M., Hughes J.M., Broadhurst M.K., Dibattista J.D., Fielder S., Walburn J.W., Furlan E.M. (2022). Environmental DNA (eDNA) as a tool for assessing fish biomass: A review of approaches and future considerations for resource surveys. Environ. DNA.

[12] Pfleger M.O., Rider S.J., Johnston C.E., Janosik A.M. (2016). Saving the doomed: Using eDNA to aid in detection of rare sturgeon for conservation (Acipenseridae). Glob. Ecol. Conserv..

[13] Mizumoto H., Mitsuzuka T., Araki H. (2020). An environmental DNA survey on distribution of an endangered salmonid species, *Parahucho perryi*, in Hokkaido, Japan. Front. Ecol. Evol..

[14] Yu D., Shen Z.Y., Chang T., Li S., Liu H.Z. (2021). Using environmental DNA methods to improve detectability in an endangered sturgeon (*Acipenser sinensis*) monitoring program. BMC Ecol. Evol..

[15] Xu N., Zhu B., Shi F., Shao K., Que Y.F., Li W.T., Li W., Jiao W.J., Tian H., Xu D.M. (2018). Monitoring seasonal distribution of an endangered anadromous sturgeon in a large river using environmental DNA. Sci. Nat..

[16] Spear M.J., Embke H.S., Krysan P.J., Zanden M.J.V. (2021). Application of eDNA as a tool for assessing fish population abundance. Environ. DNA.

[17] Xin Y., Guo Y., Sun M.J., Yu G., Ma Z.H., Pei K., Qin C.X. (2022). Optimal conditions to quantify the relationship between eDNA concentration and biomass in *Acanthopagrus latus*. Water.

[18] Pont D., Rocle M., Valentini A., Civade R., Jean P., Maire A., Roset N., Schabuss M., Zornig H., Dejean T. (2018). Environmental DNA reveals quantitative patterns of fish biodiversity in large rivers despite its downstream transportation. Sci. Rep..

[19] Zhang J.M., Ding R.Y., Wang Y.R., Wen J.T. (2022). Experimental study on the response relationship between environmental DNA concentration and biomass of *Schizothorax prenanti* in still water. Front. Ecol. Evol..

[20] Takahara T., Minamoto T., Yamanaka H., Doi H., Kawabata Z.I. (2012). Estimation of fish biomass using environmental DNA. PLoS ONE.

[21] Itakura H., Wakiya R., Yamamoto S., Kaifu K., Sato T., Minamoto T. (2019). Environmental DNA analysis reveals the spatial distribution, abundance, and biomass of Japanese eels at the river-basin scale. Aquat. Conserv..

[22] Doi H., Katano I., Sakata Y., Rio S., Toshihiro K., Mariko N., Kousuke I., Koki Y., Koji T. (2017). Detection of an endangered aquatic heteropteran using environmental DNA in a wetland ecosystem. R. Soc. Open Sci..

[23] Shogren A.J., Tank J.L., Egan S.P., Diogo B., Tenna R. (2019). Riverine distribution of mussel environmental DNA reflects a balance among density, transport, and removal processes. Freshw. Biol..

[24] Karlsson E., Ogonowski M., Sundblad G., Sundin J., Svensson O., Nousiainen I., Vasemägi A. (2022). Strong positive relationships between eDNA concentrations and biomass in juvenile and adult pike (*Esox lucius*) under controlled conditions: Implications for monitoring. Environ. DNA.

[25] Sint D., Kolp B., Rennstam R.O., Füreder L., Traugott M. (2022). The amount of environmental DNA increases with freshwater crayfish density and over time. Environ. DNA.

[26] Minamoto T., Fukuda M., Katsuhara K.R., Fujiwara A., Hidaka S., Yamamoto S., Takahashi K., Masuda R. (2017). Environmental DNA reflects spatial and temporal jellyfish distribution. PLoS ONE.

[27] Jerde C.L., Chadderton W.L., Mahon A.R., Renshaw M.A., Corush J., Budny M.L., Mysorekar S., Lodge D.M. (2013). Detection of Asian carp DNA as part of a great lakes basin-wide surveillance program. Can. J. Fish. Aquat. Sci..

[28] Benoit N.P., Robinson K.M., Kellogg C.T.E., Lemay M.A., Hunt B.P.V. (2023). Using qPCR of environmental DNA (eDNA) to estimate the biomass of juvenile Pacific salmon (*Oncorhynchus* spp.). Environ. DNA.

[29] Mauvisseau Q., Parrondo M., Fernández M.P., García L., Martínez J.L., García-Vázquez E., Borrell Y.J. (2017). On the way for detecting and quantifying elusive species in the sea: The *Octopus vulgaris* case study. Fish. Res..

[30] Gao T.X., Chen Z., Wang X.Y., Zhang H.B., Shi H.L. (2022). Correlation between the density of cultured *Sebastiscus marmoratus* and its environmental DNA. Chin. J. Hydrobiol..

[31] Lacoursière-Roussel A., Rosabal M., Bernatchez L. (2016). Estimating fish abundance and biomass from eDNA concentrations: Variability among capture methods and environmental conditions. Mol. Ecol. Resour..

[32] Stoeckle M.Y., Adolf J., Charlop-Powers Z., Dunton K.J., Hinks G., VanMorter S.M. (2021). Trawl and eDNA assessment of marine fish diversity, seasonality, and relative abundance in coastal New Jersey, USA. ICES J. Mar. Sci..

[33] Hansen B.K., Bekkevold D., Clausen L.W., Nielsen E.E. (2018). The sceptical optimist: Challenges and perspectives for the application of environmental DNA in marine fisheries. Fish Fish..

[34] Stewart K.A. (2019). Understanding the effects of biotic and abiotic factors on sources of aquatic environmental DNA. Biodivers. Conserv..

[35] Goldberg C.S., Strickler K.M., Pilliod D.S. (2015). Moving environmental DNA methods from concept to practice for monitoring aquatic macroorganisms. Biol. Conserv..

[36] Pilliod D.S., Goldberg C.S., Arkle R.S., Waits L.P. (2013). Estimating occupancy and abundance of stream amphibians using environmental DNA from filtered water samples. Can. J. Fish. Aquat. Sci..

[37] Baldigo B.P., Sporn L.A., George S.D., Ball J.A. (2017). Efficacy of environmental DNA to detect and quantify brook trout populations in headwater streams of the Adirondack Mountains, New York. Trans. Am. Fish. Soc..

[38] Yamamoto S., Minami K., Fukaya K., Takahashi K., Sawada H., Murakami H., Tsuji S., Hashizume H., Kubonaga S., Horiuchi T. (2016). Environmental DNA as a ‘snapshot’of fish distribution: A case study of Japanese jack mackerel in Maizuru Bay, Sea of Japan. PLoS ONE.

[39] Peltier H., Dabin W., Daniel P., Canneyt O.V., Dorémus G., Huon M., Ridoux V. (2012). The significance of stranding data as indicators of cetacean populations at sea: Modelling the drift of cetacean carcasses. Ecol. Indic..

[40] Li M., Shan X.J., Wang W.J., Ding X.S., Dai F.G., Lü D., Wu H.H. (2020). Studying the retention time of *Fenneropenaeus chinensis* eDNA in water. Prog. Fish. Sci..

[41] Rees H.C., Maddison B.C., Middleditch D.J., Patmore J.R.M., Gough K.C. (2014). The detection of aquatic animal species using environmental DNA–a review of eDNA as a survey tool in ecology. J. Appl. Ecol..

[42] Roussel J.M., Paillisson J.M., Treguier A., Petit E. (2015). The downside of eDNA as a survey tool in water bodies. J. Appl. Ecol..

[43] Barnes M.A., Turner C.R. (2016). The ecology of environmental DNA and implications for conservation genetics. Conserv. Genet..

[44] Geerts A.N., Boets P., Van den Heede S., Goethals P., Van der Heyden C. (2018). A search for standardized protocols to detect alien invasive crayfish based on environmental DNA (eDNA): A lab and field evaluation. Ecol. Indic..

[45] Barnes M.A., Turner C.R., Jerde C.L., Renshaw M.A., Chadderton W.L., Lodge D.M. (2014). Environmental conditions influence eDNA persistence in aquatic systems. Environ. Sci. Technol..

[46] Goldberg C.S., Sepulveda A., Ray A., Baumgardt J., Waits L.P. (2013). Environmental DNA as a new method for early detection of New Zealand mudsnails (*Potamopyrgus antipodarum*). Freshw. Sci..

[47] Cerco C.F., Schultz M.T., Noel M.R., Skahill B., Kim S.C. (2018). A fate and transport model for Asian carp environmental DNA in the Chicago area waterways system. J. Great Lakes Res..

[48] Strickler K.M., Fremier A.K., Goldberg C.S. (2015). Quantifying effects of UV-B, temperature, and pH on eDNA degradation in aquatic microcosms. Biol. Conserv..

[49] Yan H.G., Dong Z.L., Ma T.T., Zhang L.B., Wang X.Y., Ye H., Yao W.Z., He W.P. (2020). Detection and biomass assessment of *Procypris rabaudi* based on environmental DNA. J. Fish. China.

[50] Wood S.A., Biessy L., Latchford J.L., Zaiko A., von Ammon U., Audrezet F., Cristescu M.E., Pochon X. (2020). Release and degradation of environmental DNA and RNA in a marine system. Sci. Total Environ..

[51] Rice C.J., Larson E.R., Taylor C.A. (2018). Environmental DNA detects a rare large river crayfish but with little relation to local abundance. Freshw. Biol..

